# A case for expanding carrier testing to include actionable X‐linked disorders

**DOI:** 10.1002/ccr3.1806

**Published:** 2018-09-19

**Authors:** Alan F. Rope, Tia L. Kauffman, Pat Himes, Laura M. Amendola, Sumit Punj, Yassmine Akkari, Amiee Potter, James V. Davis, Jennifer L. Schneider, Jacob A. Reiss, Mari J. Gilmore, Carmit K. McMullen, Deborah A. Nickerson, C. Sue Richards, Gail P. Jarvik, Benjamin S. Wilfond, Katrina A. B. Goddard

**Affiliations:** ^1^ Department of Genetics Kaiser Permanente Northwest Portland Oregon; ^2^ Center for Health Research Kaiser Permanente Northwest Portland Oregon; ^3^ Department of Medicine Division of Medical Genetics University of Washington Seattle Washington; ^4^ Department of Molecular and Medical Genetics Oregon Health & Science University Portland Oregon; ^5^ Department of Genome Sciences University of Washington Seattle Washington; ^6^ Treuman Katz Center for Pediatric Bioethics Seattle Children's Hospital and Research Institute Seattle Washington; ^7^ Divison of Bioethics Department of Pediatrics University of Washington School of Medicine Seattle Washington

**Keywords:** expanded carrier screening, informed decision making, X‐linked

## Abstract

A research study utilizing whole‐genome sequence analysis for preconception carrier screening provided a genome‐first detection of a severe de novo Factor VIII mutation in a woman with implications for pregnancy management and life‐saving interventions of her newborn son, and a challenge to the existing paradigm regarding carrier testing.

## 1 INTRODUCTION

Population carrier screening traditionally has targeted specific autosomal recessive or X‐linked conditions that have a relatively higher prevalence in at least some populations. However, as the cost of clinical genomic sequencing continues to decline, there is potential to use this approach, most often utilized for identifying a molecular etiology for a clinical phenotype, for expanded carrier screening.

As part of the Clinical Sequencing Exploratory Research (CSER) consortium, we investigated the use of genome sequencing for clinical preconception carrier screening in a cohort of healthy, not‐yet‐pregnant women to determine carrier status for variants in 728 genes related to autosomal recessive, X‐linked, and mitochondrial conditions.^1^ This study was reviewed and approved by the Kaiser Permanente Northwest Institutional Review Board. All participants provided written consent. During this study, we identified a pathogenic *F8* variant in a woman with no previously recognized signs or family history of hemophilia, which quickly became one of the more intriguing developments from this project.

## 2 CASE REPORT

**Figure 1:**
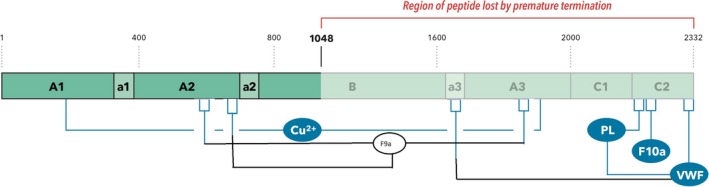
The c.3144G>A (p.Trp1048*) would eliminate a region of the gene product, which is important in the trafficking of the protein from the endoplasmic reticulum to the Golgi apparatus and would predictably disrupt interactions with von Willebrand factor, cell membrane phospholipids, activated Factors IX and X and the copper‐binding domain.

The study participant was a 34 year old who had three first trimester miscarriages without an identified etiology. She enrolled in the study and subsequently became pregnant prior to receiving her genome sequencing test results. At 15 weeks of gestation, she was reported to carry a novel pathogenic variant in *F8,* c.3144G>A (p.Trp1048*). Pathogenic variants in *F8* are associated with X‐linked recessive hemophilia A. This variant is predicted to result in an early termination codon and shortened protein, consistent with severe hemophilia A in a male, and no or mild phenotype in a female carrier (Figure 1).

After receiving this result, the participant was referred to medical genetics to discuss pregnancy management and prenatal diagnostic testing. During her debriefing interview, a few weeks after she learned the results, she expressed feeling very emotional and surprised given there was no known history of this condition in her family and the fact that previous prenatal genetic testing had revealed “no red flags” for her or her husband. However, she also expressed gratitude for having the information and ability to now map out a pregnancy care plan. “The fact that it was an X‐linked issue came out of the blue! It was definitely not what I was expecting. If it is a boy, at least we know our treatment plans at this point in my pregnancy…if we hadn't done this, we wouldn't have known and the risk would have been high anyway.”

The couple was referred to the regional hemophilia center at an academic‐based hospital. They were able to attend a conference for families affected by hemophilia and requested ultrasonography to determine the sex of the fetus, which was male. They declined amniocentesis at that time due to the risk of possible associated complications to the fetus and because they had decided to continue with the pregnancy, regardless of what the fetal testing would show.

At 33 weeks of gestation, when the possibility of preterm delivery lessened the odds of complications to the baby, the couple scheduled amniocentesis and prenatal testing so that they could be informed and best prepared for the delivery and management after delivery. Amniocentesis was performed shortly after and the results were disclosed at 35 weeks of gestation. The testing indicated that her male fetus was carrying the pathogenic *F8* variant.

At 37 weeks and 5 days of gestation, the expectant mother was admitted and underwent a low transverse incision with delivery of a viable, macrosomic male. The extraction was described as difficult. Birthweight: 4320 g, head circumference: 36.0 cm, length: 51.2 cm, with Apgar scores of 9 and 9 at 1 and 5 minutes, respectively.

As per the birth plan, the neonatal care team was vigilant for any potential birth‐related sequelae of factor VIII deficiency. Within 24 hours, the infant became irritable, pale, and developed downward trending blood pressures, with a mean arterial pressures dropping to the high 20s. He was re‐examined and noted to have an open, bulging anterior fontanel, with diffuse bogginess across the scalp, and a head circumference measuring 39 cm, due to what was ultimately determined to be a subgaleal hemorrhage by head ultrasonography.

An umbilical venous catheter was placed and the baby was immediately transfused with a normal saline bolus, antihemophilic factor/von Willebrand factor complex, packed red blood cells, and platelets. Hematologic studies before these interventions showed a hemoglobin of 7.3 g/dL (15.0‐24.6 g/dL), a hematocrit of 20.4% (50.0‐62.0%), and factor VIII activity at <0.03 units/mL (0.60‐1.60 units/mL).

He received additional transfusions and was started on a continuous infusion of antihemophilic factor/von Willebrand factor complex along with boluses to titrate factor VIII levels between the range of 50% and 100%. Given the magnitude of the hemorrhage, it was anticipated that he would be at increased risk of hyperbilirubinemia. As such, phototherapy was initiated and his unconjugated bilirubin peaked at 18.7 at 1 week of life, just prior to discharge.

To facilitate receiving infusion therapy on a regular basis, a catheter port was placed at 5 months of age and his course has been without further complications. He is regularly followed through the Hemophilia Center and is an otherwise a healthy 16‐month‐old with normal growth and development.

## 3 DISCUSSION

This case illustrates how genomic approaches to expanded carrier screening can impact perinatal care by identifying pathogenic variants in individuals who do not know they are at risk of having a child with a genetic disorder. The risk of an affected child is dramatically higher when the finding is associated with a female carrying an X‐linked recessive condition, as 25% of offspring are predicted to be affected. In contrast, for autosomal recessive conditions, the father must also contribute a pathogenic variant in order to reach 25% risk. Hemophilia A is an X‐linked disorder characterized by deficiency in factor VIII activity due to pathogenic variants of *F8*. This condition can result in a coagulopathy with the possibility of high morbidity when not properly managed. Males with severe factor VIII deficiency are usually diagnosed during the neonatal period through birth‐related trauma or neonatal‐related procedures.^2^ Molecular studies estimate that at least 30% of newly diagnosed cases of hemophilia occur because of a de novo variant and thus without a positive family history.^3^ While carrier females may also be at risk of bleeding, particularly in the case of skewed X‐inactivation, the severity of their disease is usually comparable to what is seen in mildly affected males. As in our case, variants leading to new stop codons and frameshift variants are also associated with a severe phenotype.

Awareness of the disorder in the neonatal care unit allowed for rapid detection and treatment of the subgaleal hemorrhage. A subgaleal hemorrhage occurs when the emissary veins between the skull and the intracranial venous sinuses are sheared or torn, and blood collects in the subgaleal space. This region is located between the galea aponeurotica and the periosteum of the skull. This subgaleal space covers the entire surface of cranium and has the potential to contain a pool in excess of 250 mL of fluid. A term newborn's blood volume is estimated to be approximately 80 mL/kg.^4^ As such, these conditions are serious and potentially fatal.

Overall, subgaleal hemorrhages are rare, with an incidence of four per 10,000, with higher rates, up to 59 per 10,000, for vacuum‐assisted or forceps extractions.^5^ Other recognized risk factors for subgaleal hemorrhage include primiparous mothers, term infants when compared to preterm infants, and infants with underlying coagulopathies. The mortality rate is between 12% and 25%.^4^ Neonatal subgaleal hemorrhage may be the presenting sign of hemophilia A.^6^

The knowledge of identifying a severe, potentially life‐threatening genetic variant provided the opportunity for a successful medical outcome. The research participant and her partner made informed decisions that would best protect their son's health. These included careful monitoring of the mother's factor VIII status during the pregnancy, deferring diagnostic amniocentesis to avoid the possible sequelae of preterm delivery, electing to deliver by cesarean section and before 38 weeks of gestation to avoid the risks associated with a vaginal delivery, and advanced preparation of the perinatal care team to monitor for and act upon bleeding associated with this condition.

**Figure 2:**
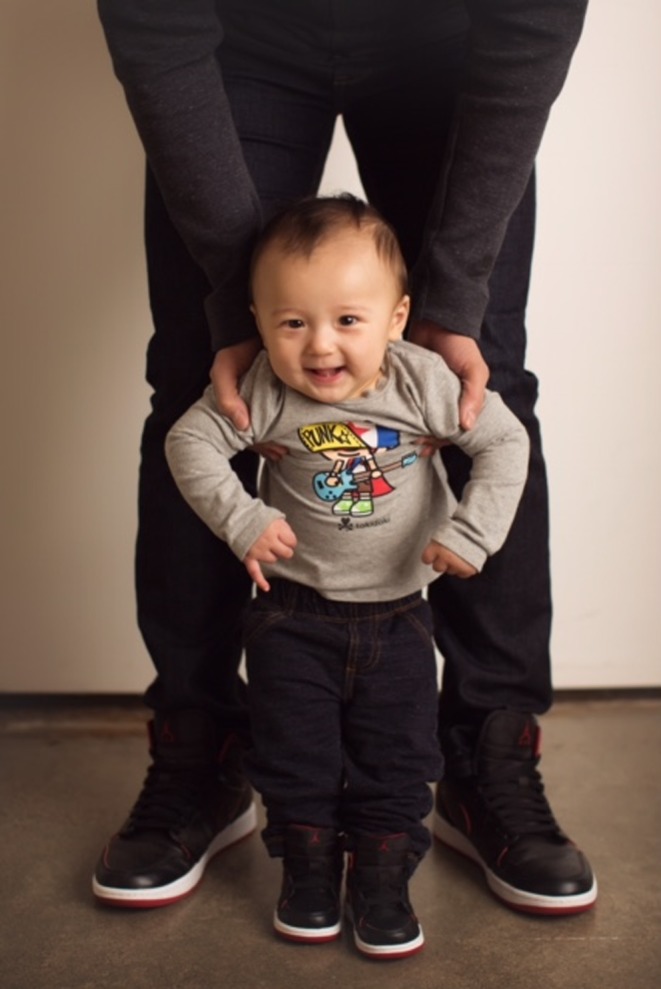
Research participant's affected son at 7 months of age. Copyright 2017, Click by Suzanne.

Without having identified the maternal carrier status for the severe *F8* variant, the outcome of this case almost certainly would have been worse and may have resulted in fatal complications for the research participant's son. This report is an important illustration of how genomic approaches to expanded carrier screening can have a direct impact on perinatal care. Though not discussed in this report, our study did not identify any recessive conditions that would have influenced medical management in the prenatal or in early childhood periods. In contrast, three X‐linked pathogenic mutations were identified in mothers without a family history, all of whom coincidentally went on to conceive males.^7^ Other X‐linked conditions with similar high risk of an affected child, such as severe combined immunodeficiency, hemophilia B, adrenoleukodystrophy, agammaglobulinemia, and Lesch‐Nyhan and Hunter syndromes, for which interventions may be available, could be detected similarly and therefore we are advocating for carrier testing to include medically actionable X‐linked conditions. While newborn screening using genomic approaches offers similar potential, it is important to note two differences. First, given the delay in the disclosure of newborn screening results, prenatal detections can offer better opportunities for intervention in the immediate neonatal period. In addition, preconception or prenatal detection offers the parents opportunities for deliberative decision‐making and psychosocial preparation that may also include elective termination of an affected pregnancy or preimplantation genetic diagnosis as part of in vitro fertilization (Figure 2).

## CONFLICT OF INTEREST

None declared.

## AUTHOR CONTRIBUTION

Rope: reviewed the clinical care of the patient, drafted the initial manuscript, reviewed and revised the manuscript. Kauffman: designed the study, collected data from the participant, reviewed and revised the manuscript. Himes: provided genetic counseling to the participant and reviewed and revised the manuscript. Amendola, Davis, Schneider, Reiss, Gilmore, and McMullen: contributed to study design, reviewed and revised the manuscript. Punj, Akkari, and Potter: conducted laboratory analyses, reviewed and revised the manuscript. Nickerson, Richards, and Jarvik: conceptualized and designed the study, conducted laboratory analyses, and reviewed and revised the manuscript. Goddard and Wilfond: conceptualized and designed the study, drafted, reviewed, and revised the manuscript.
